# Targeted next generation sequencing of parotid gland cancer uncovers genetic heterogeneity

**DOI:** 10.18632/oncotarget.4015

**Published:** 2015-05-25

**Authors:** Inga Grünewald, Claudia Vollbrecht, Jeannine Meinrath, Moritz F. Meyer, Lukas C. Heukamp, Uta Drebber, Alexander Quaas, Dirk Beutner, Karl-Bernd Hüttenbrink, Eva Wardelmann, Wolfgang Hartmann, Reinhard Büttner, Margarete Odenthal, Markus Stenner

**Affiliations:** ^1^ Institute of Pathology, University Hospital of Cologne, Cologne, Germany; ^2^ Department of Pathology, University Hospital of Muenster, Muenster, Germany; ^3^ Department of Otorhinolaryngology, Head and Neck Surgery, University Hospital of Cologne, Cologne, Germany; ^4^ Center for Molecular Medicine Cologne, University of Cologne, Cologne, Germany; ^5^ Department of Otorhinolaryngology, Head and Neck Surgery, University Hospital of Muenster, Muenster, Germany

**Keywords:** salivary gland cancer, individualized therapy, PIK3CA, HRAS, carcinogenesis

## Abstract

Salivary gland cancer represents a heterogeneous group of malignant tumors. Due to their low incidence and the existence of multiple morphologically defined subtypes, these tumors are still poorly understood with regard to their molecular pathogenesis and therapeutically relevant genetic alterations.

Performing a systematic and comprehensive study covering 13 subtypes of salivary gland cancer, next generation sequencing was done on 84 tissue samples of parotid gland cancer using multiplex PCR for enrichment of cancer related gene loci covering hotspots of 46 cancer genes.

Mutations were identified in 22 different genes. The most frequent alterations affected *TP53*, followed by *RAS* genes, *PIK3CA*, *SMAD4* and members of the *ERB* family. *HRAS* mutations accounted for more than 90% of *RAS* mutations, occurring especially in epithelial-myoepithelial carcinomas and salivary duct carcinomas. Additional mutations in *PIK3CA* also affected particularly epithelial-myoepithelial carcinomas and salivary duct carcinomas, occurring simultaneously with *HRAS* mutations in almost all cases, pointing to an unknown and therapeutically relevant molecular constellation. Interestingly, 14% of tumors revealed mutations in surface growth factor receptor genes including *ALK*, *HER2, ERBB4, FGFR, cMET* and *RET,* which might prove to be targetable by new therapeutic agents. 6% of tumors revealed mutations in *SMAD4*.

In summary, our data provide novel insight into the fundamental molecular heterogeneity of salivary gland cancer, relevant in terms of tumor classification and the establishment of targeted therapeutic concepts.

## INTRODUCTION

Salivary gland carcinomas (SGC) are rare malignant tumors accounting for 6% of head and neck cancers and 0.3% of all human malignancies. The majority of SGC occurs in the parotid gland [1]. The current World Health Organization (WHO) tumor classification recognizes 24 different salivary gland carcinoma subtypes that are characterized by highly variable biological behavior [1, 2]. Due to their low incidence and their broad histological and clinical diversity, diagnosis and therapy of these tumors are challenging. Primary therapy usually comprises surgery and/or radiotherapy, whereas conventional chemotherapy is mostly employed with a palliative aim in recurrent or metastatic disease. However, limited clinical trial data exist on systemic therapeutic approaches in SGC, and standardized targeted therapies are currently not available [2, 3].

In the past few years, multiple therapeutically relevant genetic alterations in tumors of other organs have been described, and appropriate targeted therapies have been integrated in treatment protocols. The most striking examples include the tyrosine kinase inhibitor treatment of *EGFR*-mutated pulmonary adenocarcinomas [4, 5], EGFR-directed treatment strategies in colorectal adenocarcinomas wild-type for *KRAS* and *NRAS* [6], adjuvant or neoadjuvant treatment of gastrointestinal stromal tumors carrying mutations of *c-KIT* or *PDGFRA* [7] and BRAF inhibitor treatment in BRAF V600E mutated malignant melanomas [8].

Recently developed high through-put, next generation parallel sequencing technologies offer the opportunity of sensitive detection and quantification of genetic alterations. Employment of next generation sequencing (NGS) has started to become an interesting alternative to conventional sequencing approaches in the identification of the genetic background of cancer through genome-wide association studies. Prospectively, NGS will therefore serve as an important tool for the molecular characterization of cancer for diagnostic, prognostic and predictive purposes through the identification of characteristic patterns of mutations, parts of them probably indicating options for targeted therapeutic approaches [9–11].

Because of their low incidence and great heterogeneity, knowledge on the molecular pathogenesis and therapeutically relevant genetic alterations in SGC is currently still very limited. The recent identification of recurrent chromosomal translocations in some common subtypes of SGC represents important advances in the understanding of the molecular pathogenesis of SGC. These findings provide biomarkers for molecular diagnostics and may, in the long term, help in the development of new individualized therapeutic strategies [3, 12].

Apart from few previous studies which either focused on sub-entities as adenoid cystic carcinoma [13, 14] or salivary duct carcinoma [15] or which were based on a limited number of analyzed genes [16], systematic large-scale sequencing approaches in SGC have not been performed yet. The present study was therefore intended to elucidate genetic mechanisms of the molecular pathogenesis of SGC and to identify potential therapeutically applicable genetic alterations in a large collection of SGC covering all major histological subtypes.

## RESULTS

Next generation sequencing was performed on 84 tumor tissue samples from which sufficient DNA could be extracted. Clinicopathological characteristics of these 84 patients are summarized in Table 1.

**Table 1:** Patients' characteristics

Patients' characteristics	*N* (%)
**Patients**	84
Male	42 (50.0%)
Female	42 (50.0%)
**Age (years)**
Mean ± SD	58.8 ± 18.1
Median	61
Minimum/Maximum	16/89
**Resection margins**
R0	49 (58.3%)
R1	16 (19.0%)
R2	4 (4.8%)
Rx	15 (17.9%)
**pT-stage**	
pTx	4 (4.8%)
pT1	14 (16.7%)
pT2	20 (23.8%)
pT3	16 (19.0%)
pT4a	25 (29.8%)
pT4b	4 (4.8%)
pT4	1 (1.2%)
**pN-stage**	
pNx	9 (10.7%)
pN0	44 (52.4%)
pN1	9 (10.7%)
pN2	20 (23.8%)
pN3	2 (2.4%)
**Extracapsular spread**	
Unknown	7 (8.3%)
Yes	13 (15.5%)
No	64 (76.2%)
**M-stage**	
Mx	3 (3.6%)
M0	73 (86.9%)
M1	8 (9.5%)
**Lymphangiosis**	
Unknown	6 (7.1%)
Yes	14 (16.7%)
No	64 (76.2%)
**Hemangiosis**	
Unknown	6 (7.1%)
Yes	14 (16.7%)
No	64 (76.2%)
**Perineural invasion**	
Unknown	7 (8.3%)
Yes	26 (31.0%)
No	51 (60.7%)
**Type of parotidectomy**	
Lateral	6 (7.1%)
Total	48 (57.1%)
Radical	26 (31.0%)
Subtotal	4 (4.8%)
**Neck dissection**	
Yes	77 (91.7%)
No	7 (8.3%)

**Figure 1:**
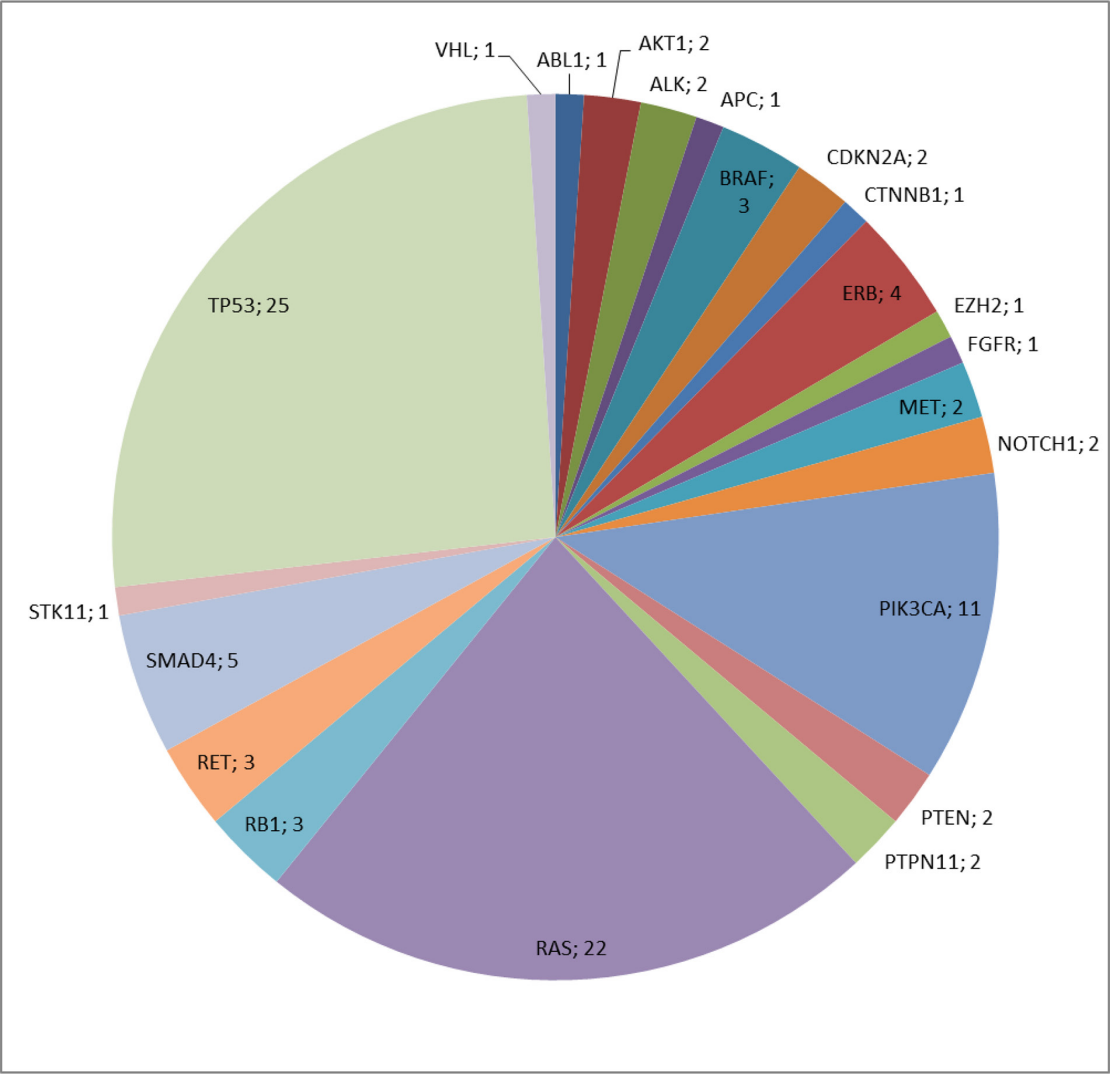
Absolute frequency of mutations in SGC

In the analyzed parotid gland carcinomas mutations were identified in 22 different genes out of 46 cancer genes covered by the Ion AmpliSeq™ Cancer Panel. Many tumors revealed several mutations in different genes, occasionally more than one mutation was found in the same gene. In 35 tumors (42%), no mutations were detected. The absolute frequencies of detected mutations are demonstrated in Figure 1. The most frequent alterations comprise mutations in *TP53*, followed by *RAS* genes, *PIK3CA*, *SMAD4* and members of the *ERB* family.

**Figure 2:**
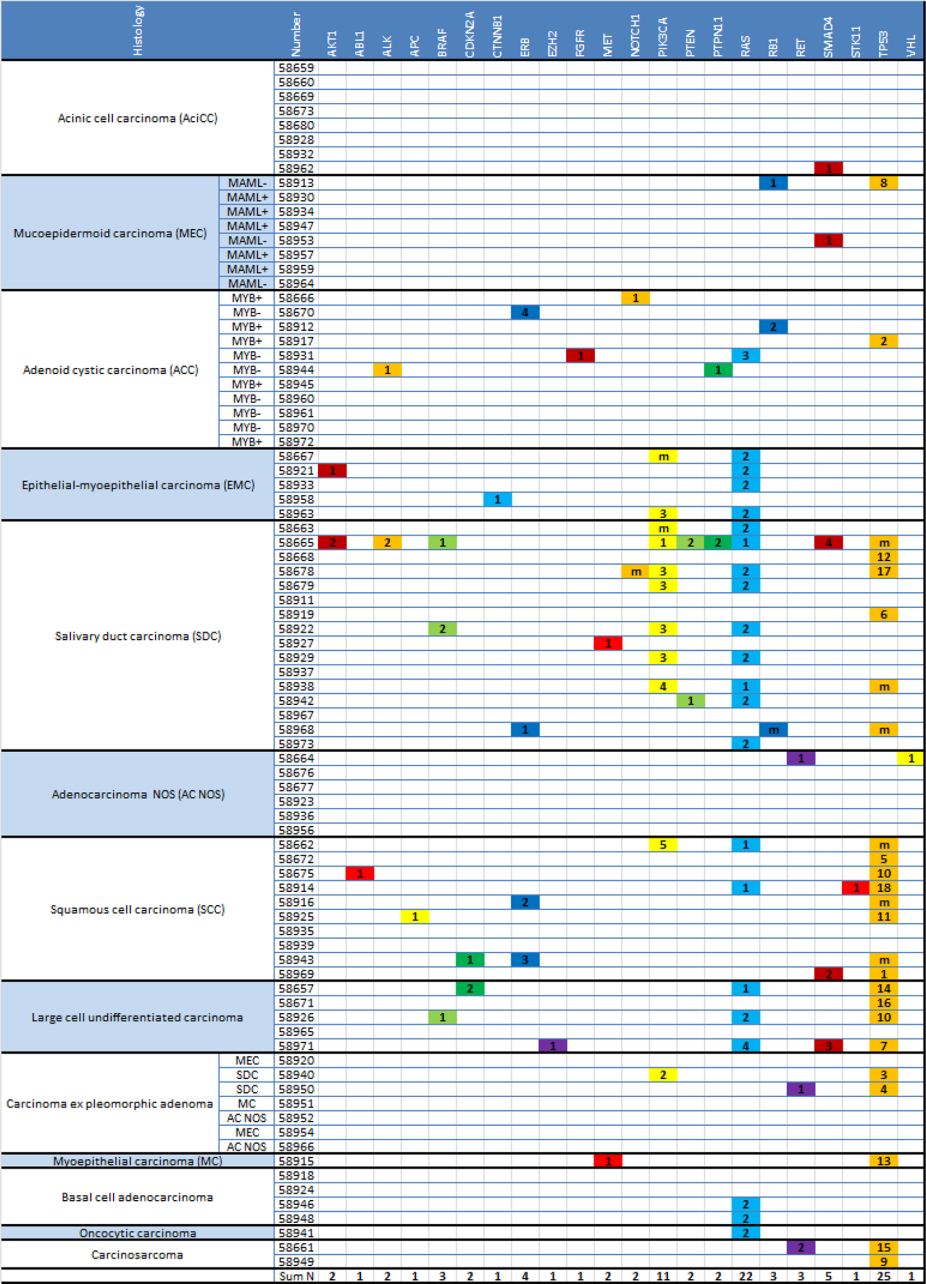
Mutational status in subtypes of SGC. Detected mutations are displayed for each gene/gene family and each tumor sample, sorted by histological subtype (see Table 2 for key of mutation numbers) In MEC and ACC translocation status is specified (MAML−/+: MAML translocation negative/positive; MYB−/+: MYB translocation negative/positive).

In Figure 2 all detected mutations are displayed for each analyzed tumor sample according to the different gene families. Cases are sorted by histological tumor type. The key for mutation numbers is displayed in Table 2.

**Table 2:** 

**Table 2: A: Key for mutation numbers in Figure 1**	
Gene	Mutation number
ABL1	1: Exon 5 p.D295N (c.882_883delinsAA)
AKT1	1: Exon 4 p.E17K (c.49G>A) 2: Exon 4 p.G33D (c.98G>A)
ALK	1: Exon 23 p.F1174L (c.3522C>A) 2: Exon 23 p.M1199I (c.3597G>A)
APC	1: Exon 14 p.E868K (c.2602G>A)
BRAF	1: Exon 11 p.G466E/p.G466V (c.1397G>A/c.1397G>T) 2: Exon 15 p.V600E (c.1799T>A)
CDKN2A	1: Exon 3 p.R80* (c.238C>T) 2: Exon 3 p.R58* (c.172C>T)
CTNNB1	1: Exon 3 p.I35T (c.104T>C)
ERB	1: ERBB2 Exon 24 p.T862A (c.2584A>G) 2: ERBB4 Exon 6 p.G240R (c.718G>A) 3: ERBB4 Exon 8 p.K312R (c.935A>G) 4: ERBB4 Exon 8 p.S303F (c.908C>T)
EZH2	1: Exon 16 p.Y646H (c.1936T>C)
FGFR	1: FGFR2 Exon 10 p.N550K (c.1650T>A) 2: FGFR3 Exon 7 p.D270N (c.808G>A)
MET	1: Exon 2 p.E168D (c.504G>T)
NOTCH1	1: Exon 26 p.P1581_P1582del (c.4741_4746delCCGCCG) m: multiple
PIK3CA	1: Exon 10 p.E545K (c.1633G>A) 2: Exon 14 p.E707K (c.2119G>A) 3: Exon 21 p.H1047L/p.H1047R (c.3140A>T/c.3140A>G) 4: Exon 5 p.N345K (c.1035T>A) 5: Exon 7 p.V409L (c.1225G>C) m: multiple
PTEN	1: Exon 7 p.D252G (c.755A>G) 2: Exon 5 p.G132D (c.395G>A)
PTPN11	1: Exon 13 p.T507K (c.1520C>A) 2: Exon 13 p.V490I (c.1468G>A)
RAS	1: HRAS Exon 2 p.G12D/p.G13D/p.G13R (c.35G>A/c.38G>A/c.37G>C) 2: HRAS Exon 3 p.Q61K/p.Q61L/p.Q61R (c.181C>A/c.182A>T/c.182A>G) 3: NRAS Exon 3 p.A59D (c.176C>A) 4: NRAS Exon 3 p.Q61L (c.182A>T)
RB1	1: Exon 11 e11-1 (c.1050_splice) 2: Exon 18 e18-1 (c.1696_splice) m: multiple
RET	1: Exon 13 p.R770* (c.2307_2308delinsTT) 2: Exon 15 p.S904L (c.2711_2712delinsTG)
SMAD4	1: Exon 9 p.M331I (c.993G>A) 2: Exon 12 p.P522L (c.1565C>T) 3: Exon 8 p.Q311* (c.931C>T) 4: Exon 5 p.G176E (c.527G>A)
STK11	1: Exon 8 p.F354L (c.1062C>G)
TP53	1: Exon 7 p.C229fs (c.686_687delGT) 2: Exon 8 p.E298* (c.892G>T) 3: Exon 6 p.H214R (c.641A>G) 4: Exon 7 p.N239S (c.716A>G) 5: Exon 8 p.P278S (c.832C>T) 6: Exon 4 p.Q100* (c.298C>T) 7: Exon 6 p.R213* (c.637C>T) 8: Exon 8 p.R267fs (c.801delG) 9: Exon 10 p.R333fs (c.997_998insC) 10: Exon 7 p.S241F/p.S241Y (c.722C>T/c.722C>A) 11: Exon 6 p.V197M/p.V197R (c.589G>A/c.589_590delinsAG) 12: Exon 4 p.Y103* (c.309C>A) 13: Exon 6 p.Y220C (c.659A>G) 14: Exon 8 p.D281N (c.841G>A) 15: Exon 8 p.R273L (c.818G>T) 16: Exon 5 p.M133fs (c.397delA) 17: Exon 8 p.V274fs (c.819_820insT) 18: Exon 7 p.R248G (c.742C>G) m: multiple
VHL	1: Exon 1 p.R107C (c.319C>T)

**Table 2: B: Cases with multiple mutations in one gene (indicated by “m” in Figure 1)**	
Case	Mutations in tde same gene
58667	PIK3CA: Exon 21 p.G1049R (c.3145G>C), Exon 2 p.K111E (c.331A>G)
58663	PIK3CA: Exon 7 p.V409L (c.1225G>C), Exon 10 p.E545K (c.1633G>A)
58665	TP53: Exon 2 p.P13L (c.38C>T), Exon 4 p.P87L (c.260C>T), Exon 5 p.R158H (c.473G>A), Exon 6 p.P223L (c.668C>T), Exon 10 p.G361R (c.1081G>A)
58678	NOTCH1: Exon 2 p.G1572D (c.4715G>A), p.V1578del (c.4732_4734delGTG)
58938	TP53: Exon 8 p.R273L (c.818G>T), p.F270S (c.809T>C)
58968	RB1: Exon 4 p.Y155* (c.465T>A), Exon 10 p.L335* (c.1004T>A) TP53: Exon 7 p.G245V (c.734G>T), Exon 10 p.G360A (c.1079G>C)
58662	TP53: Exon 4 p.Q104* (c.310C>T), Exon 7 p.C242Y (c.725G>A)
58916	TP53: Exon 4 p.S94fs (c.282_331delATCTTCTGTCCCTTCCCAGAAAACCTACCAGGGCAGCTACGGTTTCCGTC), Exon 8 p.E298* (c.892G>T)
58943	TP53: Exon 5 p.C135fs (c.403delT), Exon 6 p.R196P (c.587G>C)

The most frequent genetic alterations occurred in salivary duct carcinomas (SDC), large cell undifferentiated carcinomas, squamous cell carcinomas (SCC) and epithelial-myoepithelial carcinomas (EMC) whereas acinic cell carcinomas (AciCC), mucoepidermoid carcinomas (MEC), adenoid cystic carcinomas (ACC), adenocarcinomas NOS and basal cell adenocarcinomas were found to carry only few mutations.

**Figure 3:**
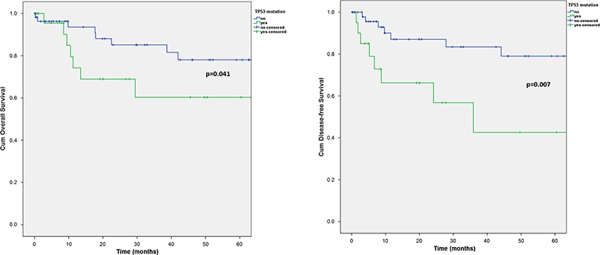
Kaplan-Meier chart of overall A. and disease-free B. survival according to the TP53 mutational status

Nearly 30% of SGC, mainly the more aggressive subtypes, showed mutations in *TP53* and more than 7% carried more than one mutation in the gene. Tumors with *TP53* mutations displayed a significant worse overall (OS) and disease-free survival (DFS) (5-year OS with *TP53* mutation: 60.3%, 5-year OS without *TP53* mutation: 78.0%, *p* = 0.041; 5-year DFS with *TP53* mutation: 42.6%, 5-year DFS without *TP53* mutation: 79.0%; *p* = 0.007) (Figure 3). In general there was a preponderance of more aggressive tumor subtypes in the group with *TP53* mutations. Interestingly, in the subgroups of AciCC, EMC, adenocarcinomas NOS and basal cell adenocarcinomas, *TP53* mutations did not occur.

26% of all SGC contained *RAS* mutations. Interestingly, only very few mutations were detected in *NRAS* with absence of mutations in *KRAS*, but more than 90% of *RAS* mutations affecting *HRAS*. Among the *HRAS* mutations, 75% were substitutions at codon 61, only 25% of cases showed substitutions at codons 12/13. SDC and EMC are most often affected by *RAS* and especially *HRAS* mutations at codon 61. Thus, among EMC, four of the five cases showed a *HRAS* mutation and among the 16 SDC cases, *HRAS* mutations occurred in 9 tumors. However, in these subgroups no statistically significant differences of overall survival were detectable referring to *RAS* mutational status. On the other hand, *RAS* mutations did not occur in AciCC, MEC, adenocarcinomas NOS and in carcinomas ex pleomorphic adenomas.

*PIK3CA* mutations most frequently occurred in SDC and were found in nearly 44% of SDC. Four mutations affected the hotspot in the kinase domain in codon 1047, two mutations occurred at the hotspot of the helicase domain (codon 545), and one tumor showed a mutation in codon 345, which is not located in a hotspot region but which has been described in other tumors according to COSMIC database [13, 17]. One SDC that occurred as a carcinoma ex pleomorphic adenoma carried a rare non-hotspot *PIK3CA* mutation in codon 707, which has been previously described in a papillary carcinoma of the breast [18]. Additionally, two cases (40%) of EMC showed *PIK3CA* mutations, with one tumor carrying two simultaneous *PIK3CA* mutations in codon 1049 and in codon 111, both having been described before [19, 20]. Interestingly, *PIK3CA* mutations occurred almost always in combination with *HRAS* mutations (*p* < 0.0001), only one case of carcinoma ex pleomorphic adenoma (SDC) carried a *PIK3CA* mutation without a simultaneous *HRAS* mutation. As for *RAS* mutations, no statistically significant differences of overall survival were detectable in the subgroups of SDC and EMC referring to *PIK3CA* mutational status.

As alternative hits in the PI3K/AKT signaling pathway, one case of EMC without *PIK3CA* mutation exhibited a mutation in *AKT1* in codon 17, a well-known and often described mutation [21, 22], and one case of SDC without *PIK3CA* mutation carried a *PTEN* mutation in codon 252, another known mutation [23, 24]. Furthermore, one case of SDC with *PIK3CA* mutation showed simultaneous *AKT1* and *PTEN* mutations. One case of SDC carried a BRAF V600E mutation in addition to *PIK3CA* and *HRAS* mutations.

In about 14% of SGC mutations in surface growth factor receptor genes *ALK*, *HER2, ERBB4, FGFR, cMET* and *RET* were detected. Mutations in effectors of the RAS/RAF/MAPK cascade and the PI3K/AKT signaling pathway occurred almost exclusively in tumors with wild-type growth factor receptors; only one case with a *FGFR2* mutation showed a simultaneous *NRAS* mutation and another case with an *ALK* mutation carried several simultaneous mutations in *AKT1*, *BRAF*, *PIK3CA*, *PTEN* and *HRAS*.

6% of tumors revealed mutations in *SMAD4*. However, no clustering of *SMAD4* mutations occurred in a special histological subtype.

## DISCUSSION

Here we present a parallel sequencing analysis of 46 cancer related genes on a collection of 84 parotid gland carcinomas comprising the major histological subtypes. Apart from few previous studies which either focused on sub-entities as ACC [13, 14] or SDC [15] or which were based on a limited number of analyzed genes [16] our work represents the first systematic large scale sequencing analysis in salivary gland carcinomas.

All in all, with our systematic approach on 84 tumor samples covering the histological heterogeneity we detected somatic gene mutations in 58% of the samples involving 22 different genes. Although some loci were only moderately covered which might be due to long storage of tissues and low quality of DNA, extracted from formalin fixed and paraffin embedded tissues, variants with low coverage and low frequency were successfully validated by conventional Sanger sequencing (Supplementary Figures S1, S2).

A previous less systematic report based on a significantly smaller gene panel and a less representative tumor cohort (with 70% of the analyzed samples representing four tumor types) suggested a mutational rate of 25% only [16]. Generally, compared to the other SGC entities, AciCC, MEC and ACC revealed a low frequency of mutations pointing to other mechanisms in tumorigenesis than specific mutational alterations. Accordingly, previous studies of ACC reported a low rate of somatic mutations [13, 14, 16]. This is in line with the finding of recurrent chromosomal translocations in MEC and ACC involving the *MAML2* and *MYB* genes, respectively, which appear to play a key role in the molecular pathogenesis of these neoplasms [3, 12]. The resulting fusion proteins appear to be functionally essential as pathogenic drivers in these tumors. Interestingly, in MEC the few detected mutations occurred only in MAML translocation negative tumors, whereas in ACC single mutations were detected in MYB translocation negative as well as MYB translocation positive tumors. For AciCC, very little is known about the genetic profile; no gene fusions or recurrent mutations have been identified yet [3]. Recently the PI3K/AKT/mTOR signaling pathway has been shown to be activated in AciCC in immunohistochemical studies [16, 25]. In agreement with the results presented by Cros and colleagues [16] our data suggest that this pathway activation does preferentially not result from genomic mutations in PI3K/AKT/mTOR key effectors as *AKT1*, *PIK3CA* or *PTEN*.

As the most recurrent finding, occurring predominantly in the more aggressive subtypes, 30% of the analyzed tumors displayed one or several mutations in the *TP53* gene. This finding is in good agreement with the data presented by Ku and colleagues who reported a high rate of p53 mutations in SDC [15]. While not representing a strong diagnostic marker, this finding provides relevant translational information by excluding almost one third of SGC from a potential therapy with small-molecule inhibitors of MDM2, which are currently evaluated in clinical studies in other solid tumors [26].

The second most common genetic alteration observed in our study were mutations in *RAS* oncogenes. The three closely related RAS proteins (HRAS, KRAS, NRAS) promote oncogenesis when mutated at codon 12, 13 or 61 [27]. Lung and colon adenocarcinomas show *KRAS* mutations in a subset of tumors, particularly in codon 12 and 13, and these tumors harboring *KRAS* mutation are associated with resistance to anti-EGFR directed therapeutic approaches [28, 29]. In a small percentage of colon carcinomas *NRAS* mutations are detectable, which, unlike *KRAS*, mostly occur in codon 61 [30]. In our set of tumors, *KRAS* mutations were not detected at all whereas 90% of *RAS* mutations occurred in *HRAS*, affecting codon 61 in 75%. Accordingly, previous studies in SGC demonstrated a very low rate of *KRAS* mutations [31] as well as a comparatively high rate of *HRAS* mutations, particularly occurring in EMC and involving codon 61 [16, 32]. Consistently, data from the COSMIC database indicate an overall *HRAS* mutation rate of 19% in parotid gland carcinomas (http://cancer.sanger.ac.uk/cosmic/browse/tissue#sn=salivary_gland&ss=parotid&hn=carcinoma&sh=&in=t&src=tissue). Our comparative overview on *RAS* mutations in SGC confirms the finding of frequent *HRAS* mutations in EMC, however, compared to previous studies, *HRAS* mutation frequency in EMC was found to be much higher than estimated before and appears to reach 80% (4/5) [16, 32]. Further, we detected *HRAS* mutations in a high percentage of SDC (9/16), a biologically and therapeutically highly relevant finding, which has not been described before: considering potential therapeutic approaches in SGC targeting surface growth factor receptors as EGFR or HER2 have to take into account the high frequency of *RAS* mutations known to cause primary resistance.

As mentioned above, the PI3K/AKT/mTOR pathway has recently been shown to be implicated in salivary gland tumorigenesis [15, 16, 25, 33, 34]. In this respect two previous studies showed *PIK3CA* mutations in 20% and 30% of analyzed SDC, respectively [33, 34]. We confirm frequent *PIK3CA* mutations in SDC with a frequency of almost 44% (7/16). Additionally, 40% (2/5) of EMC showed *PIK3CA* mutations. *PTEN* or *AKT1* mutations as detected in individual cases of SDC appear to represent a different mechanism of PI3K/AKT/mTOR pathway activation, with simultaneous mutations in *PTEN* and/or *AKT1* and *PIK3CA* obviously occurring in individual cases. These findings imply the major role of PI3K/AKT/mTOR signaling in SDC and EMC tumorigenesis and offer the opportunity for an individualized cancer therapy targeting PI3K/AKT/mTOR pathway effectors. This idea is supported by a recent case report on successful treatment of two patients with SDC with temsirolimus, an inhibitor of the mTOR pathway [35].

Interestingly, any tumor but one with *PIK3CA* mutation additionally carried simultaneous *HRAS* mutations pointing to the parallel activation of the two major receptor tyrosine kinase downstream signaling pathways, a finding not reflected in previous studies. In the traditional concepts of molecular tumorigenesis parallel activation of strong oncogenic signaling pathways is an unusual finding, however, recently, co-existence of activating mutations in the MAPK and PI3K pathways have been shown in tumors of other organs such as colon carcinoma [36, 37]. This finding, as well, provides interesting insights into SDC biology and carries relevant implications for targeted tumor therapy indicating the need to simultaneously block both pathways in order to obtain a tumor response. Additionally, in single cases the BRAF V600E mutation appears to occur in SGC, providing the possibility of a targeted therapy with BRAF inhibitor therapy in individual patients [8].

Activating mutations in surface growth factor receptor genes also seem to play a role in SGC tumorigenesis as about 14% of SGC showed mutations in genes as *ALK*, *HER2, ERBB4, FGFR, cMET* and *RET*. In two SDC without *PIK3CA* and *HRAS* mutation, mutations in *HER2* and *cMET* occurred, respectively, and one SDC ex pleomorphic adenoma showed a *RET* mutation, pointing to alternative mechanisms in tumorigenesis of this tumor entity beyond *PIK3CA* and *HRAS* mutations. Individual cases of SGC reveal mutations in *ALK*, *cMET* or *RET* that might be responsive to targeted therapies with molecularly directed agents. *ALK* mutated tumors can be targeted with ALK-inhibitors as crizotinib, providing potential treatment options also in *ALK* mutated SGC. One case in our set showed an *ALK* mutation in codon 1174 that is associated with resistance to crizotinib implying the need of treatment with the new, second-generation ALK-inhibitor ceritinib [38, 39]. There is also some evidence that *cMET* mutated tumors can be affected by molecular agents [40]. *RET* mutated tumors can accordingly be targeted by RET-directed therapeutic agents as vandetanib, pointing to further treatment options in SGC [41].

In line with previous studies, the group of ACC does not show any *cKIT* mutation [42], and despite cKIT overexpression in over 90% of ACC, treatment studies with cKIT-directed approaches with imatinib revealed no clinical benefit [43]. *cKIT* is one of multiple MYB target genes, and as oncogenic *MYB* translocations appear to play an important role in ACC tumorigenesis, inhibitors of MYB itself or more than one MYB target might be promising in ACC [3].

Almost 6% of tumors in our study carried mutations in *SMAD4*. SMAD4 acts as a signal transducer protein in the TGFβ pathway that plays an important role in the control of cell growth and carcinogenesis [44]. *SMAD4* was previously shown to be involved in pancreatic cancer tumorigenesis [45]. However, we could not observe a characteristic accumulation of *SMAD4* mutations in a special histological subtype, but mutations were scattered over different tumor types. In accordance with another study [46], *SMAD4* therefore appears to play a minor role in salivary gland carcinogenesis.

With regard to the biological properties of the tumors making up the category “adenocarcinoma NOS“, the low frequency of mutational events detected in this group of tumors was unexpected. However, the consistent finding of a mutational pattern involving *p53*, *PIK3CA* and *HRAS* which is in good agreement with data presented before [15] in the group of SDC but not in the adenocarcinoma NOS cases further indirectly validates our diagnostic classification. Consequent application of the diagnostic criteria of SDC effectively groups together these high-grade tumors in the SDC category and delineates them from other subgroups. We hypothesize that adenocarcinoma NOS, if classified appropriately, represents an assembly of molecularly distinct sub-entities, the scarcity of classic oncogene and tumor suppressor gene mutations pointing to the presence of major and characteristic genetic events, e.g. gene fusions as recently identified in the newly established entity mammary analogue secretory carcinoma of the salivary glands molecularly defined by an ETV6-NTRK3 fusion.

In this NGS approach on SGC of different histological subtypes we provide the first systematic and representative comparative overview on the genetic landscape of SGC. Beyond the confirmation of previously reported mutations in *PIK3CA* and *HRAS* in subsets of SGC, we show that these mutations often occur simultaneously and that other oncogenic effectors are of pathogenic relevance in SGC tumorigenesis and classification. In the future, comprehensive mutational analysis of SGC will crucially contribute to a molecular based reclassification of these cancer entities. The low incidence of salivary gland carcinomas requires the use of long time stored paraffin embedded archive tissue samples with low DNA quality for studies as the present, and we here successfully document the technical feasibility of such approaches. Future prospective studies should be designed to enclose larger cohorts of tumors treated in a standardized manner.

## MATERIALS AND METHODS

### Ethics statement

Investigation has been conducted in accordance with the ethical standards and according to the Declaration of Helsinki and according to national and international guidelines and has been approved by the authors' institutional review board.

### Patient data and specimens

The retrospective study included 112 patients with newly diagnosed parotid gland cancer treated at the Department of Otorhinolaryngology, Head and Neck Surgery at the University Hospital of Cologne between 1998 and 2011. All patients were treated by primary definitive surgery and potential adjuvant radiation according to patients' cancer stage. Tumor staging was adapted to the 7^th^ edition of the UICC TNM classification for carcinomas of the salivary glands. Patients were followed up at the outpatients department at periodic visits in 3 to 6 months. At the time of analysis, 20 patients had died and 31 patients had developed a histologically confirmed relapse. Mean follow-up time was 40.0 months (range 0 to 269). The study was approved by the local Ethics Committee of the University Hospital of Cologne (No. 13–265).

Formalin-fixed and paraffin-embedded (FFPE) material of the patients was obtained from the archive of the Department of Pathology at the University Hospital of Cologne. All tumors were independently re-evaluated by two experienced pathologists (IG and WH) with regard to histopathological diagnosis in accordance with WHO 2005 classification of tumors of salivary glands. FISH analyses for MYB and MAML translocations were performed as described previously [47, 48] using locus-specific break-apart probes (ZytoVision GmbH, Bremerhaven, Germany) and were considered in the differential diagnosis of tumor entities as appropriate. Tumors with break-apart signals in at least 20% of tumor cells were assumed as translocation positive. 45.5% (5/11) of adenoid cystic carcinomas and 62.5% (5/8) of mucoepidermoid carcinomas showed MYB or MAML translocations, respectively (Figure 2). For the partially difficult differential diagnosis of SDC and adenocarcinoma NOS a diagnostic algorithm based on a characteristic „ductal“ growth pattern and the expression of the androgen receptor was employed [49, 50]. Consistent with data published before, HER2 positivity, either by immuohistochemistry or by FISH, was detectable in a large subset of SDC but not in the tumors categorized as adenocarcinoma NOS [15]. The somewhat controversial diagnosis of primary SCC of salivary glands, which is, however, included in the WHO 2005 classification of tumors was only made after systematic exclusion of any other primarius by thorough otorhinolaryngological endoscopy, CT scans including the neck region, thorax and abdomen and a dermatological examination. Since no other primarius was detectable, these cases were included in this study as primary parotid SCC according to the WHO 2005 classification. With regard to histomorphological criteria, only cases with evident infiltrating tumor growth within salivary gland parenchyma were included.

Finally, sufficient DNA from 84 cases was obtained to conduct NGS.

The collection of SGC included 13 different histological tumor types, the frequencies are shown in Table 3.

**Table 3:** Histological tumor types of SGC included in the study

Histological tumor type	*N*	%
Acinic cell carcinoma	8	9.5
Mucoepidermoid carcinoma	8	9.5
Adenoid cystic carcinoma	11	13.1
Epithelial-myoepithelial carcinoma	5	6.0
Salivary duct carcinoma	16	19.0
Adenocarcinoma NOS	6	7.1
Squamous cell carcinoma	10	11.9
Larg cell undifferentiated carcinoma	5	6.0
Carcinoma ex pleomorphic adenoma	7	8.3
Myoepithelial carcinoma	1	1.2
Basal cell adenocarcinoma	4	4.8
Oncocytic carcinoma	1	1.2
Carcinosarcoma	2	2.4
Total	84	100.0

### Tumor macrodissection and DNA extraction

Sections were prepared from FFPE material and stained with hematoxylin & eosin (H&E). Six additional sections of 6 μm thickness were cut, mounted onto glass slides and used for macrodissection. In total, 1 cm^2^ tumor area corresponding to the tumor area of H&E-stained section were scraped off with a scalpel and collected into plastic tubes.

Subsequently, the DNA was automatically extracted using the Maxwell DNA FFPE isolation kit on a Maxwell platform (Promega GmbH, Mannheim, Germany) according to the manufacturer's instructions.

### DNA quality control and quantification

DNA quality and quantity was assessed by gel electrophoresis and fluorescence absorbance (QuantiFluor dsDNA system, Promega). In order to quantify the amplifiable DNA real time PCR was performed using the *HFE* gene as amplifying reference (234bp). Standard curves were prepared from unmutated high quality DNA isolated from native human embryonic kidney cells (HEK-293) in a range of 0.195 to 50 ng. Real-time PCR was carried out in triplicates with 1 μl DNA, each, in a 20 μl reaction mix containing 0.4 μM of the HFE forward and reverse primer (Supplementary Table S1 A) and the GoTaq® qPCR Master Mix (Promega).

### NGS library construction by multiplex PCR

In order to selectively amplify cancer related hotspot regions, the primer sets of the Ion AmpliSeq™ *Cancer Hotspot Panel* v2 (Life Technologies, Carlsbad, CA, USA) were used. In total, the panel contained 207 amplicons (Supplementary Table S1 B), covering hotspots of 46 genes. 10 ng of amplifiable DNA was applied to multiplex PCR by means of Ion AmpliSeq™ Library Kit 2.0 (Life Technologies) following the manufacturer's instructions. After target enrichment, DNA was purified from half of the reaction volume. All purification and size selection steps were performed with Agencourt® AMPure® XP magnetic beads (Beckman Coulter, Inc., Brea, CA, USA) using the robotic Biomek® FX^p^ workstation (Beckman Coulter, Inc.). Subsequently, PCR enriched DNA was adenylated and ligated to NEXTflex™ DNA barcodes - 48 (Bioo Scientific, Austin, Texas, USA) in 30 μl assays containing the T4-Ligase and the Switch solution of the Ion AmpliSeq™ Kit 2.0 from Life Technologies. After additional purification and size selection steps, targeted DNA was enriched by 10 PCR cycles, each with a 15 seconds denaturation and 30 seconds 60°C annealing and elongation step using the NEXTflex™ primer mix (Bioo Scientific) and the Platinum® PCR SuperMix High Fidelity polymerase (Ion AmpliSeq™ Kit 2.0, Life Technologies).

Finally, the quality of enriched targets was evaluated by microfluidic based electrophoresis using High Sensitivity DNA Kit on a 2100 Bioanalyzer (Agilent Technologies, Santa Clara, CA, USA). Following, library quantification was performed by qPCR using library adapter specific primer sets (Supplementary Table S1 C) and dilution series of PhiX Control V3 (Illumina, Inc., San Diego, Ca, USA) as a standard curve. For sequencing, samples were pooled in an equimolar ratio. 15 pM library pools including 2.5 % PhiX Control V3 were prepared for sequencing according to the MiSeq System User Guide (Illumina, Inc.). Finally, sequencing was carried out on a MiSeq instrument (Illumina, Inc.) using the v2 chemistry as recommended by the manufacturer.

### Data analysis and statistics

Fastq files were generated by the MiSeq Reporter Software (Illumina, Inc.) and analysed by an in-house developed bioinformatics pipeline based on our general cancer genome analysis algorithm [51] which was further optimized for the diagnostic workflow as described by König *et al*. [52]. Identified variants were then filtered as follows: From the total of variants, all variants found only in one sequencing direction were eliminated. Next, silent variants, hotspot artefacts and reading errors were filtered, which are recognized by high occurrence in the sample set and quite constant frequency. In addition, putative false positive variants, shown to be located in a sequence region with high background noise by integrative genomic viewer analysis (https://www.broadinstitute.org/igv/) were deleted. In addition, germline SNP and all variants below 4% were filtered, ending up with variants listed in Supplementary Table S2 and Table 2. From these identified variants, in particular, variants with low read numbers or low frequency were validated by Sanger sequencing (Supplementary Table S2, cases marked in grey were confirmed by Sanger sequencing, and Supplementary Figures S1 and S2).

The variant impact on the protein function was assessed by the MutationAssessor (http://mutationassessor.org; release 2) [53] (Supplementary Table S2). For statistical analysis, the IBM SPSS Statistics 22 software (SPSS Inc., Chicago, IL, USA) was applied and Kaplan-Meier survival analysis and Log rank test were performed. The association between experimental findings was analyzed using χ^2^-test for categorical data. Nominal two-sided *p*-values are reported. The significance level was set at *p* < 0.05.

### Conventional Sanger sequencing

Conventional Sanger sequencing for a methodologically independent validation of subsets of mutations was carried out according to standard procedures using the BigDye® Terminator v3.1 Cycle Sequencing Kit (Life Technologies) and the primer sets shown in Supplementary Table S1D.

## SUPPLEMENTARY FIGURES AND TABLES


