# Supplementary material for: Targeted next generation sequencing of parotid gland cancer uncovers genetic heterogeneity
Source: Oncotarget. 2015 May 25;6(20):18224–37. doi: 10.18632/oncotarget.4015 (PMC4627247; doi:10.18632/oncotarget.4015)
Supplement: Supplementary file 1 [file oncotarget-06-18224-s001.pdf]

SUPPLEMENTARY FIGURES AND TABLES

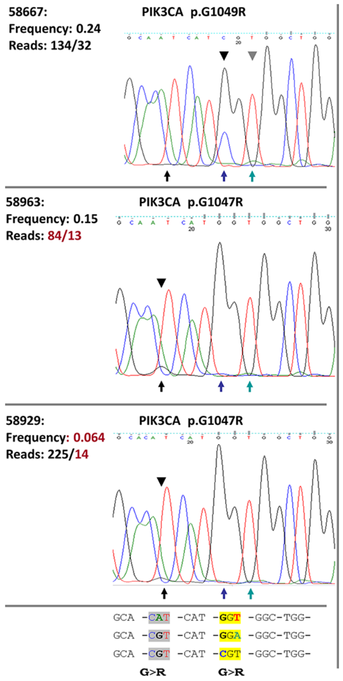

Supplementary Figure S1: Sanger validation of PIK3CA variants with low frequency. The PIK3CA pG1049R and the pG1047R variants of 24, 15 or 6.4% frequency were validated by conventional Sanger sequencing. Arrows indicate the loci of interest. Comparison of the labeled loci demonstrates that even the PIK3CA p.G1047R variant of sample 58929 was validated, though its frequency was lower than 10% by NGS. The coverage is presented by the ratio of the total to the mutant read number. Details of low NGS frequency or NGS coverage are indicated by red text.

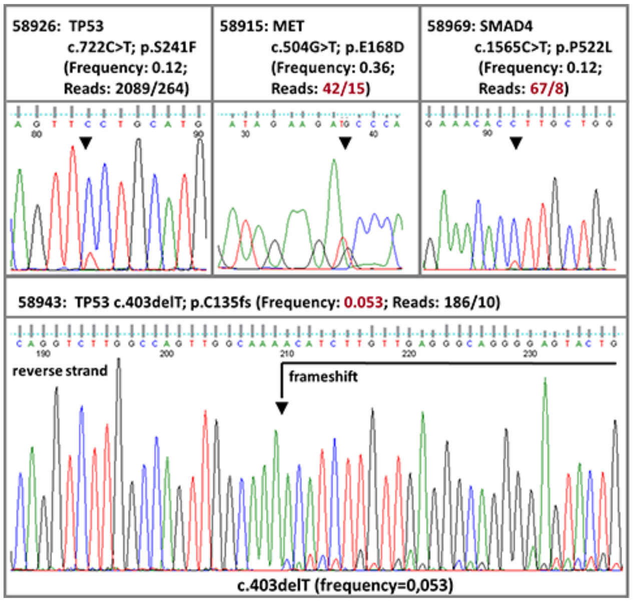

Supplementary Figure S2: Sanger validation of variants with low frequency or low coverage. The TP53 pS241F and the SMAD pP522L mutation occurring with NGS frequency of about 12% were also identified by conventional Sanger sequencing. Though coverage was under 100 reads per locus, the pE168D mutation in the MET gene and pP522L mutation in the SMAD4 gene was detected. Interestingly, also the frame shift (fs) mutation pC135fs, predefined to occur with 5.3% frequency by NGS, was detected by Sanger sequencing. The coverage is presented by the ratio of the total to the mutant read number. Low NGS frequency or NGS coverage are indicated by red text.

Supplementary Table S1: Primers Used for PCR Assays

A. HFE Primers Used for DNA Input Quantification

Name	Sequence	AT
HFE forward	5′ ATG GAT GCC AAG GAG TTC GAA CC	60°C
HFE reverse	5 ′GCC ATA ATT ACC TCC TCA GGC AC	60°C

B. Primer Coordinates Used for Library Construction by means of Multiplex PCR

Gene	Targeted HG19_coordinates	Transcript ID
ABL1_exon4	chr9:133738294-133738378	NM_007313
ABL1_exon5	chr9:133747442-133747530	
ABL1_exon6	chr9:133748279-133748417	
ABL1_exon7	chr9:133750307-133750405	
AKT1_exon3	chr14:105241433-105241519	NM_001014431
AKT1_exon6	chr14:105246445-105246583	
ALK_exon23	chr2:29432572-29432680	NM_004304
ALK_exon25	chr2:29443607-29443729	
APC_exon17	chr5:112173871112173962	NM_001127510
APC_exon17	chr5:112174557-112174666	
APC_exon17	chr5:112175143-112175268	
APC_exon17	chr5:112175315-112175443	
APC_exon17	chr5:112175567-112175703	
APC_exon17	chr5:112175740-112175862	
APC_exon17	chr5:112175920-112176035	
ATM_exon8	chr11:108117765-108117865	NM_000051
ATM_exon9	chr11:108119815-108119891	
ATM_exon11	chr11:108123515-108123618	
ATM_exon17	chr11:108137931-108138025	
ATM_exon26	chr11:108155083-108155180	
ATM_exon34	chr11:108170456-108170556	
ATM_exon35	chr11:108172362-108172467	
ATM_exon36	chr11:108173630-108173703	
ATM_exon39	chr11:108180902-108180960	
ATM_exon50	chr11:108200915-108200993	
ATM_exon54	chr11:108204634-108204684	
ATM_exon55	chr11:108205731-108205816	
ATM_exon56	chr11:108206523-108206628	
ATM_exon59	chr11:108218015-108218144	
ATM_exon61	chr11:108225549-108225632	

(Continued)

Gene	Targeted HG19_coordinates	Transcript ID
ATM_exon63	chr11:108236042-108236140	
ATM_exon63	chr11:108236186-108236285	
BRAF_exon11	chr7:140453102-140453221	NM_004333
BRAF_exon15	chr7:140481391-140481515	
CDH1_exon3	chr16:68835602-68835697	NM_004360
CDH1_exon8	chr16:68846024-68846151	
CDH1_exon9	chr16:68847199-68847302	
CDKN2A_exon2	chr9:21970940-21971066	NM_001195132
CDKN2A_exon2	chr9:21971090-21971219	
CSF1R_exon7	chr5:149433593-149433692	NM_005211
CSF1R_exon22	chr5:149452991-149453073	
CTNNB1_exon3	chr3:41266029-41266147	NM_001904
EGFR_exon3	chr7:55211044-55211126	NM_005228
EGFR_exon7	chr7:55221792-55221919	
EGFR_exon15	chr7:55232962-55233053	
EGFR_exon18	chr7:55241635-55241729	
EGFR_exon19	chr7:55242411-55242540	
EGFR_exon20	chr7:55248965-55249090	
EGFR_exon20	chr7:55249122-55249245	
EGFR_exon21	chr7:55259507-55259628	
ERBB2_exon19	chr17:37880212-37880340	NM_004448
ERBB2_exon20	chr17:37880953-37881061	
ERBB2_exon21	chr17:37881324-37881453	
ERBB4_exon3	chr2:212288904-212288990	NM_005235
ERBB4_exon4	chr2:212530051-212530180	
ERBB4_exon6	chr2:212576799-212576910	
ERBB4_exon7	chr2:212578288-212578415	
ERBB4_exon8	chr2:212587133-212587239	
ERBB4_exon9	chr2:212589764-212589867	
ERBB4_exon15	chr2:212652719-212652806	
ERBB4_exon23	chr2:212812075-212812169	
EZH2_exon16	chr7:148508706-148508791	NM_004456
FBXW7_exon5	chr4:153245410-153245492	NM_033632
FBXW7_exon8	chr4:153247277-153247369	
FBXW7_exon9	chr4:153249355-153249477	
FBXW7_exon10	chr4:153250852-153250926	
FBXW7_exon11	chr4:153258901-153259023	

(*Continued*)

Gene	Targeted HG19_coordinates	Transcript ID
FGFR1_exon5	chr8:38282140-38282254	NM_001174067
FGFR1_exon8	chr8:38285851-38285975	
FGFR2_exon7	chr10:123257952-123258045	NM_022970
FGFR2_exon7	chr10:123274721-123274835	
FGFR2_exon9	chr10:123279417-123279544	
FGFR2_exon12	chr10:123279607-123279713	
FGFR3_exon7	chr4:1803551-1803653	NM_01163213
FGFR3_exon9	chr4:1806081-1806187	
FGFR3_exon14	chr4:1807833-1807930	
FGFR3_exon16	chr4:1808311-1808399	
FGFR3_exon18	chr4:1808881-1809006	
FLT3_exon11	chr13:28592579-28592663	NM_004119
FLT3_exon14	chr13:28602275-28602379	
FLT3_exon16	chr13:28608227-28608348	
FLT3_exon20	chr13:28610093-28610184	
GNA11_exon5	chr19:3118881-3118973	NM_002067
GNAQ_exon5	chr9:80409375-80409498	NM_002072
GNAS_exon8	chr20:57484396-57484504	NM_080425
GNAS_exon9	chr20:57484562-57484672	
HNF1A_exon3	chr12:121431371-121431459	NM_000545
HNF1A_exon4	chr12:121432010-121432099	
HRAS_exon2	chr11:533812-533930	NM_001130442
HRAS_exon3	chr11:534220-534308	
IDH1_exon4	chr2:209113103-209113206	NM_005896
IDH2_exon4	chr15:90631824-90631954	NM_002168
JAK2_exon14	chr9:5073729-5073857	NM_004927
JAK3_exon4	chr19:17945616-17945734	NM_000215
JAK3_exon13	chr19:17947986-17948074	
JAK3_exon16	chr19:17954134-17954225	
KDR_exon6	chr4:55946088-55946208	NM_002253
KDR_exon7	chr4:55946250-55946371	
KDR_exon11	chr4:55953775-55953860	
KDR_exon19	chr4:55955078-55955168	
KDR_exon21	chr4:55960976-55961059	
KDR_exon26	chr4:55962444-55962548	
KDR_exon27	chr4:55972952-55973071	
KDR_exon30	chr4:55979574-55979655	

(*Continued*)

Gene	Targeted HG19_coordinates	Transcript ID
KDR_exon30	chr4:55980238-55980359	
KIT_exon2	chr4:55561654-55561784	NM_000222
KIT_exon9	chr4:55592157-55592246	
KIT_exon10	chr4:55593417-55593513	
KIT_exon11	chr4:55593575-55593695	
KIT_exon13	chr4:55594170-55594279	
KIT_exon14	chr4:55595496-55595562	
KIT_exon15	chr4:55597436-55597524	
KIT_exon17	chr4:55599280-55599358	
KIT_exon18	chr4:55602673-55602751	
KRAS_exon2	chr12:25378549-25378658	NM_033360
KRAS_exon3	chr12:25380260-25380364	
KRAS_exon4	chr12:25398186-25398304	
MET_exon2	chr7:116339615-116339701	NM_001127500
MET_exon2	chr7:116340155-116340270	
MET_exon11	chr7:116403131-116403251	
MET_exon14	chr7:116411878-116411997	
MET_exon16	chr7:116417427-116417542	
MET_exon19	chr7:116423407-116423492	
MLH1_exon12	chr3:37067207-37067333	NM_000249
MPL_exon10	chr1:43814968-43815086	NM_005373
NOTCH1_exon26	chr9:139390764-139390885	NM_017617
NOTCH1_exon27	chr9:139397762-139397879	
NOTCH1_exon34	chr9:139399337-139399447	
NPM1_exon11	chr5:170837501-170837616	NM_002520
NRAS_exon2	chr1:115252185-115252269	NM_002524
NRAS_exon3	chr1:115256504-115256584	
NRAS_exon4	chr1:115258689-115258774	
PDGFRA_exon12	chr4:55140981-55141103	NM_006206
PDGFRA_exon14	chr4:55144101-55144195	
PDGFRA_exon15	chr4:55144538-55144653	
PDGFRA_exon18	chr4:55152024-55152154	
PIK3CA_exon2	chr3:178916775-178916881	NM_006218
PIK3CA_exon2	chr3:178916931-178917035	
PIK3CA_exon5	chr3:178921464-178921570	
PIK3CA_exon7	chr3:178927405-178927525	
PIK3CA_exon8	chr3:178927901-178927986	

(*Continued*)

Gene	Targeted HG19_coordinates	Transcript ID
PIK3CA_exon8	chr3:178928069-178928160	
PIK3CA_exon10	chr3:178936023-178936105	
PIK3CA_exon14	chr3:178938787-178938918	
PIK3CA_exon19	chr3:178947818-178947896	
PIK3CA_exon21	chr3:178951996-178952097	NM_006218
PIK3CA_exon21	chr3:178952140-178952237	
PTEN_exon1	chr10:89624207-89624300	NM_000314
PTEN_exon3	chr10:89685258-89685374	
PTEN_exon5	chr10:89692813-89692920	
PTEN_exon6	chr10:89711804-89711932	
PTEN_exon7	chr10:89717503-89717620	
PTEN_exon7	chr10:89717666-89717780	
PTEN_exon8	chr10:89720695-89720747	
PTEN_exon8	chr10:89720784-89720900	
PTPN11_exon3	chr12:112888118-112888228	NM_002834
PTPN11_exon13	chr12:112926835-112926961	
RB1_exon4	chr13:48919223-48919312	NM_000321
RB1_exon6	chr13:48923139-48923255	
RB1_exon10	chr13:48941601-48941724	
RB1_exon11	chr13:48942597-48942711	
RB1_exon14	chr13:48953753-48953874	
RB1_exon17	chr13:48955525-48955605	
RB1_exon18	chr13:49027105-49027178	
RB1_exon20	chr13:49033827-49033934	
RB1_exon21	chr13:49037846-49037932	
RB1_exon22	chr13:49039149-49039232	
RET_exon10	chr10:43609066-43609182	NM_020975
RET_exon11	chr10:43609874-43610010	
RET_exon13	chr10:43613794-43613893	
RET_exon15	chr10:43615546-43615687	
RET_exon16	chr10:43617314-43617433	
SMAD4_exon3	chr18:48575099-48575213	NM_005359
SMAD4_exon4	chr18:48575556-48575677	
SMAD4_exon5	chr18:48581190-48581302	
SMAD4_exon6	chr18:48584551-48584678	
SMAD4_exon7	chr18:48586251-48586361	
SMAD4_exon8	chr18:48591814-48591931	

(*Continued*)

Gene	Targeted HG19_coordinates	Transcript ID
SMAD4_exon9	chr18:48593399-48593519	
SMAD4_exon10	chr18:48603028-48603119	
SMAD4_exon11	chr18:48604658-48604774	
SMARCB1_exon2	chr20:24133953-24134064	NM_003073
SMARCB1_exon4	chr20:24143200-24143311	
SMARCB1_exon5	chr20:24145477-24145598	
SMARCB1_exon9	chr20:24176259-24176391	
SMO_exon3	chr7:128845063-128845188	NM_005631
SMO_exon5	chr7:128845957-128846063	
SMO_exon6	chr7:128846337-128846419	
SMO_exon9	chr7:128850269-128850363	
SMO_exon11	chr7:128851499-128851612	
SRC_exon14	chr20:36031666-36031769	NM_198291
STK11_exon1	chr19:1206977-1207104	NM_000455
STK11_exon4	chr19:1220310-1220450	
STK11_exon4	chr19:1220480-1220603	
STK11_exon6	chr19:1221236-1221332	
STK11_exon8	chr19:1223014-1223144	
TP53_exon2	chr17:7573923-7574035	NM_000546
TP53_exon4	chr17:7577015-7577151	
TP53_exon5	chr17:7577508-7577612	NM_000546
TP53_exon5	chr17:7578180-7578298	
TP53_exon6	chr17:7578352-7578483	
TP53_exon7	chr17:7578516-7578601	
TP53_exon8	chr17:7579350-7579485	
TP53_exon10	chr17:7579853-7579960	
VHL_exon1	chr3:10183765-10183854	NM_000551
VHL_exon2	chr3:10188186-10188306	
VHL_exon3	chr3:10191418-10191527	

C. Primer Set Used for PCR Quantification of Target Libraries

Name	Sequence	AT
P1 forward	5′ AAT GAT ACG GCG ACC ACC GAG ATC TAC AC	60°C
P2 reverse	5′ GCC ATA ATT ACC TCC TCA GGC AC	60°C

D. Primers used for Sanger Sequencing

Primer	Gene	Exon	Sequence
BRAF-11-F	BRAF	Exon 11	5'-CTGTTTGGCTTGACTTGACTTTTT-3'
BRAF-11-R	BRAF	Exon 11	5'-TGTCACAATGTCACCACATTACAT-3'
BRAF-15-F	BRAF	Exon 15	5'-ATGCTTGCTCTGATAGGAAAATGA-3'
BRAF-15-R	BRAF	Exon 15	5'-ATCCAGACAACTGTTCAAACT-3'
HRAS-1-F	HRAS	Exon 1	5'-AGGAGACCCTGTAGGAGGA-3'
HRAS-1-R	HRAS	Exon 1	5'-CCAGGCTCACCTCTATAGTG-3'
HRAS-2-F	HRAS	Exon 2	5'-GTCCTCCTGCAGGATTCCTA-3'
HRAS-2-R	HRAS	Exon 2	5'-GGTTCACCTGTACTGGTGG-3'
TP53-4aF	TP53	Exon 4	5'-CTGGTAAGGACAAGGGTTGG-3'
TP53-4aR	TP53	Exon 4	5'-TTCTGGGAAGGGACAGAAGA-3'
TP53-4aF	TP53	Exon 4	5'-GTCCCCGGACGATATTGAAC-3'
TP53-4bR	TP53	Exon 4	5'-GGATACGGCCAGGCATTG-3'
TP53-5F	TP53	Exon 5	5'-TGCCCTGACTTTCAACTCTGT-3'
TP53-5R	TP53	Exon 5	5'-GCAATCAGTGAGGAATCAGAGG-3'
TP53-6F	TP53	Exon 6	5'-GAGAGACGACAGGGCTGGT-3'
TP53-6R	TP53	Exon 6	5'-GGGAGGTCAAATAAGCAGCA-3'
TP53-7F	TP53	Exon 7	5'-TGCTTGCCACAGGTCTCC-3'
TP53-7R	TP53	Exon 7	5'-GGTCAGAGGCAAGCAGAGG-3'
TP53-8F	TP53	Exon 8	5'-CCTGATTTCCTTACTGCCTCTT-3'
TP53-8R	TP53	Exon 8	5'-AGGCATAACTGCACCCTTG-3'
PIK3CA-9F	PIK3CA	Exon 9	5'-TGACAAAGAACAGCTCAAAGCAA-3'
PIK3CA-9R	PIK3CA	Exon 9	5'-TTTTAGCACTTACCTGTGACTCCA-3'
PIK3CA-20F	PIK3CA	Exon 20	5'-GCAAGAGGCTTTGGAGTATTTCA-3'
PIK3CA-20R	PIK3CA	Exon 20	5'-ATGCTGTTTAATTGTGTGGAAGATC-3'
RET-13-F	RET	Exon 13	5'-TGACCTGGTATGGTCATGGA-3'
RET-13-R	RET	Exon 13	5'-GGAGAACAGGGCTGTATGGA-3'
MET-165-F	MET	Exon 2	5'-CCACAATCATACTGCTGACATAC-3'
MET-165-R	MET	Exon 3	5'-GGAAATAAGAAGAATTTATGGTATTGCC-3'
SMAD4-E9F	SMAD4	Exon 9	5'-TAGCTCTGTCAGCTGCTGGA-3'
SMAD4-E9R	SMAD4	Exon 9	5'-ATGGTATGAAGTACTTCGTCTAGG-3'

Supplementary Table S2: Complete list of non-synonymous variants ≥ 4% allelic frequency

Variants marked in grey were confirmed by Sanger sequencing

Pat. ID	Gene Name	Transcript	Chrom.	Variant type	cDNA change	Protein change	Allelic freq. (%)	Cov.	FI	TCC (%)
58675	ABL1	NM_007313	chr9	missense	c.882_883delinsAA	p.D295N	6.4	339	low	40
58665	AKT1	NM_001014431	chr14	missense	c.98G>A	p.G33D	4.4	1734	medium	70
58921	AKT1	NM_001014431	chr14	missense	c.49G>A	p.E17K	28.6	56	medium	80
58665	ALK	NM_004304	chr2	missense	c.3597G>A	p.M1199I	4.6	2910	low	70
58944	ALK	NM_004304	chr2	missense	c.3522C>A	p.F1174L	4.5	4119	neutral	40
58925	APC	NM_001127510	chr5	missense	c.2602G>A	p.E868K	16.4	643	low	80
58665	BRAF	NM_004333	chr7	missense	c.1397G>A	p.G466E	5	3186	high	70
58922	BRAF	NM_004333	chr7	missense	c.1799T>A	p.V600E	17.6	675	low	50
58926	BRAF	NM_004333	chr7	missense	c.1397G>T	p.G466V	9.9	507	high	40
58657	CDKN2A	NM001195132	chr9	nonsense	c.172C>T	p.R58*	73	799	n.d.	70
58943	CDKN2A	NM001195132	chr9	nonsense	c.238C>T	p.R80*	15	1125	n.d.	50
58958	CTNNB1	NM_001904	chr3	missense	c.104T>C	p.I35T	37.5	28	medium	70
58968	ERBB2	NM_004448	chr17	missense	c.2584A>G	p.T862A	19.2	173	neutral	50
58916	ERBB4	NM_005235	chr2	missense	c.718G>A	p.G240R	13.8	886	medium	50
58943	ERBB4	NM_005235	chr2	missense	c.935A>G	p.K312R	6.3	4398	medium	50
58971	EZH2	NM_004456	chr7	missense	c.1936T>C	p.Y646H	11.5	553	n.d.	80
58931	FGFR2	NM_022970	chr10	missense	c.1650T>A	p.N550K	31.6	188	neutral	80
58657	HRAS	NM_001130442	chr11	missense	c.38G>A	p.G13D	51.2	1584	medium	70
58662	HRAS	NM_001130442	chr11	missense	c.37G>C	p.G13R	44.5	990	medium	70
58663	HRAS	NM_001130442	chr11	missense	c.182A>G	p.Q61R	13.3	149	medium	30
58665	HRAS	NM_001130442	chr11	missense	c.37G>C	p.G13R	31.3	66908	medium	70
58667	HRAS	NM_001130442	chr11	missense	c.182A>G	p.Q61R	28.3	854	medium	60
58678	HRAS	NM_001130442	chr11	missense	c.182A>G	p.Q61R	36.8	364	medium	60
58679	HRAS	NM_001130442	chr11	missense	c.182A>G	p.Q61R	23.4	346	medium	30
58914	HRAS	NM_001130442	chr11	missense	c.35G>A	p.G12D	17.8	65	medium	30
58921	HRAS	NM_001130442	chr11	missense	c.182A>G	p.Q61R	33.1	130	medium	80
58922	HRAS	NM_001130442	chr11	missense	c.182A>G	p.Q61R	22.7	336	medium	50
58926	HRAS	NM_001130442	chr11	missense	c.182A>T	p.Q61L	4.5	1038	high	40
58929	HRAS	NM_001130442	chr11	missense	c.181C>A	p.Q61K	6	816	high	30
58933	HRAS	NM_001130442	chr11	missense	c.182A>G	p.Q61R	35.3	168	medium	70
58938	HRAS	NM_001130442	chr11	missense	c.37G>C	p.G13R	29.6	380	medium	40
58941	HRAS	NM_001130442	chr11	missense	c.182A>G	p.Q61R	22.3	636	medium	80
58942	HRAS	NM_001130442	chr11	missense	c.182A>G	p.Q61R	9.5	399	medium	50
58946	HRAS	NM_001130442	chr11	missense	c.182A>G	p.Q61R	41	1026	medium	50
58948	HRAS	NM_001130442	chr11	missense	c.182A>G	p.Q61R	22.8	145	medium	20
58963	HRAS	NM_001130442	chr11	missense	c.182A>G	p.Q61R	14.2	450	medium	70
58973	HRAS	NM_001130442	chr11	missense	c.182A>G	p.Q61R	30.6	76	medium	50
58915	MET	NM_001127500	chr7	missense	c.504G>T	p.E168D	36.2	42	low	70

(Continued)

Pat. ID	Gene Name	Transcript	Chrom.	Variant type	cDNA change	Protein change	Allelic freq. (%)	Cov.	FI	TCC (%)
58927	MET	NM_001127500	chr7	missense	c.504G>T	p.E168D	37.2	38	low	40
58666	NOTCH1	NM_017617	chr9	in_frame_del	c.4741_4746delCCGCCG	p.P1581_P1582del	12.4	700	medium	50
58678	NOTCH1	NM_017617	chr9	missense	c.4715G>A	p.G1572D	4.5	379	medium	60
58678	NOTCH1	NM_017617	chr9	in_frame_del	c.4732_4734delGTG	p.V1578del	4.2	382	medium	60
58931	NRAS	NM_002524	chr1	missense	c.176C>A	p.A59D	8.5	191	high	80
58971	NRAS	NM_002524	chr1	missense	c.182A>T	p.Q61L	58.1	3468	high	80
58662	PIK3CA	NM_006218	chr3	missense	c.1225G>C	p.V409L	16.6	444	medium	70
58663	PIK3CA	NM_006218	chr3	missense	c.1225G>C	p.V409L	6.1	118	medium	30
58663	PIK3CA	NM_006218	chr3	missense	c.1633G>A	p.E545K	8.5	127	low	30
58665	PIK3CA	NM_006218	chr3	missense	c.1633G>A	p.E545K	16.1	13634	low	70
58667	PIK3CA	NM_006218	chr3	missense	c.3145G>C	p.G1049R	24.2	134	low	60
58667	PIK3CA	NM_006218	chr3	missense	c.331A>G	p.K111E	20.2	218	medium	60
58678	PIK3CA	NM_006218	chr3	missense	c.3140A>T	p.H1047L	26.4	190	neutral	60
58679	PIK3CA	NM_006218	chr3	missense	c.3140A>G	p.H1047R	24.3	207	neutral	30
58922	PIK3CA	NM_006218	chr3	missense	c.3140A>G	p.H1047R	26.8	63	neutral	50
58929	PIK3CA	NM_006218	chr3	missense	c.3140A>G	p.H1047R	6.4	225	neutral	30
58938	PIK3CA	NM_006218	chr3	missense	c.1035T>A	p.N345K	18.8	635	medium	40
58940	PIK3CA	NM_006218	chr3	missense	c.2119G>A	p.E707K	5.2	207	low	60
58963	PIK3CA	NM_006218	chr3	missense	c.3140A>G	p.H1047R	14.9	84	neutral	70
58665	PTEN	NM_000314	chr10	missense	c.395G>A	p.G132D	6.7	5073	high	70
58942	PTEN	NM_000314	chr10	missense	c.755A>G	p.D252G	9.8	2570	medium	50
58665	PTPN11	NM_002834	chr12	missense	c.1468G>A	p.V490I	5.7	1428	n.d.	70
58944	PTPN11	NM_002834	chr12	missense	c.1520C>A	p.T507K	5.6	6574	n.d.	40
58912	RB1	NM_000321	chr13	splice	c.1696_splice	e18-1	10.1	98	n.d.	75
58913	RB1	NM_000321	chr13	splice	c.1050_splice	e11-1	48.3	26	n.d.	50
58968	RB1	NM_000321	chr13	nonsense	c.1004T>A	p.L335*	30.6	32	n.d.	50
58968	RB1	NM_000321	chr13	nonsense	c.465T>A	p.Y155*	55.3	34	n.d.	50
58661	RET	NM_020975	chr10	missense	c.2711_2712delinsTG	p.S904L	47.6	1058	low	80
58674	RET	NM_020975	chr10	nonsense	c.2307_2308delinsTT	p.R770*	25	1058	n.d.	70
58950	RET	NM_020975	chr10	nonsense	c.2307_2308delinsTT	p.R770*	51.9	140	n.d.	30
58665	SMAD4	NM_005359	chr18	missense	c.527G>A	p.G176E	10.9	3372	neutral	70
58953	SMAD4	NM_005359	chr18	missense	c.993G>A	p.M331I	14.5	49	low	60
58962	SMAD4	NM_005359	chr18	missense	c.993G>A	p.M331I	48.4	5698	low	80
58969	SMAD4	NM_005359	chr18	missense	c.1565C>T	p.P522L	12	67	high	70
58971	SMAD4	NM_005359	chr18	nonsense	c.931C>T	p.Q311*	42.7	198	n.d.	80
58914	STK11	NM_000455	chr19	missense	c.1062C>G	p.F354L	45.6	92	low	30
58657	TP53	NM_000546	chr17	missense	c.841G>A	p.D281N	61.1	1790	medium	70
58661	TP53	NM_000546	chr17	missense	c.818G>T	p.R273L	11.8	922	medium	80

(*Continued*)

Pat. ID	Gene Name	Transcript	Chrom.	Variant type	cDNA change	Protein change	Allelic freq. (%)	Cov.	FI	TCC (%)
58662	TP53	NM_000546	chr17	nonsense	c.310C>T	p.Q104*	20.3	412	n.d.	70
58662	TP53	NM_000546	chr17	missense	c.725G>A	p.C242Y	27.3	1356	medium	70
58665	TP53	NM_000546	chr17	missense	c.1081G>A	p.G361R	4.2	2495	neutral	70
58665	TP53	NM_000546	chr17	missense	c.260C>T	p.P87L	5.3	3134	low	70
58665	TP53	NM_000546	chr17	missense	c.38C>T	p.P13L	4.3	6164	medium	70
58665	TP53	NM_000546	chr17	missense	c.473G>A	p.R158H	4.3	18302	medium	70
58665	TP53	NM_000546	chr17	missense	c.668C>T	p.P223L	4.6	216	medium	70
58668	TP53	NM_000546	chr17	nonsense	c.309C>A	p.Y103*	43.6	274	n.d.	30
58671	TP53	NM_000546	chr17	frame_ shift_del	c.397delA	p.M133fs	72.6	1008	n.d.	70
58672	TP53	NM_000546	chr17	missense	c.832C>T	p.P278S	5.5	279	medium	15
58675	TP53	NM_000546	chr17	missense	c.722C>A	p.S241Y	5.2	1003	medium	40
58678	TP53	NM_000546	chr17	frame_ shift_ins	c.819_820insT	p.V274fs	52.3	499	n.d.	60
58913	TP53	NM_000546	chr17	frame_ shift_del	c.801delG	p.R267fs	13.1	183	n.d.	50
58914	TP53	NM_000546	chr17	missense	c.742C>G	p.R248G	4.6	252	medium	30
58915	TP53	NM_000546	chr17	missense	c.659A>G	p.Y220C	76.7	38	medium	70
58916	TP53	NM_000546	chr17	frame_ shift_del	c.282_331delATCTTCT GTCCCTTCCCAGAAA ACCTACCAGGGCAGC TACGGTTTCCGTC	p.S94fs	5.9	235	n.d.	50
58916	TP53	NM_000546	chr17	nonsense	c.892G>T	p.E298*	6.3	1020	n.d.	50
58917	TP53	NM_000546	chr17	nonsense	c.892G>T	p.E298*	9.8	691	n.d.	30
58919	TP53	NM_000546	chr17	nonsense	c.298C>T	p.Q100*	32.6	425	n.d.	50
58925	TP53	NM_000546	chr17	missense	c.589_590delinsAG	p.V197R	6.6	327	medium	80
58926	TP53	NM_000546	chr17	missense	c.722C>T	p.S241F	12.7	2089	medium	40
58938	TP53	NM_000546	chr17	missense	c.809T>C	p.F270S	14	385	medium	40
58938	TP53	NM_000546	chr17	missense	c.818G>T	p.R273L	7.5	381	medium	40
58940	TP53	NM_000546	chr17	missense	c.641A>G	p.H214R	27.8	925	medium	60
58943	TP53	NM_000546	chr17	frame_ shift_del	c.403delT	p.C135fs	5.4	186	n.d.	50
58943	TP53	NM_000546	chr17	missense	c.587G>C	p.R196P	13	4385	medium	50
58949	TP53	NM_000546	chr17	frame_ shift_ins	c.997_998insC	p.R333fs	83.3	64	n.d.	70
58950	TP53	NM_000546	chr17	missense	c.716A>G	p.N239S	13.9	603	medium	30
58968	TP53	NM_000546	chr17	missense	c.1079G>C	p.G360A	40	27	medium	50
58968	TP53	NM_000546	chr17	missense	c.734G>T	p.G245V	17.3	93	medium	50
58969	TP53	NM_000546	chr17	frame_ shift_del	c.686_687delGT	p.C229fs	26.7	412	n.d.	70
58971	TP53	NM_000546	chr17	nonsense	c.637C>T	p.R213*	48.5	1909	n.d.	80
58664	VHL	NM_000551	chr3	missense	c.319C>T	p.R107C	4.7	171	low	70

Pat. ID: Patient Identity; Chrom.: Chromosome; Allelic freq.: Allelic frequency; Cov.: Coverage; FI: Functional Impact; TCC: Tumor cell content; n.d.: not determined
